# Prison Reform

**Published:** 1886-11-20

**Authors:** 


### PRISON REFORM.

For a week, ending on the 13th instant, the National Prison Congress, with ex-President Hays as presiding officer, was in session in Atlanta. The discussions relating to the various plans of imprisoning, punishing, and reforming criminals assumed a very wide range. A number of papers were read and sermons delivered. Among these, the ablest, and, to our mind, the most practical and common-sense address was that by the Rev. Dr. Tucker. Our space will not allow us even to epitomize the many addresses made. Facts were disclosed in reference to prisons, even in our own land, so renowned for freedom and humanity, that would shock the heart of the Philanthropist to know; and it must be admitted that the great body of our law-abiding and Christian people know little or nothing of the condition of our prisons, and manifest no concern or interest in them. This is certainly wrong, and highly justifies the organization of the Prison Congress and the interest taken in it by great and good men in our country.

Rev. Dr. Haygood very truthfully and forcibly remarked that "No Government has a moral right unnecessarily to put the bodily health of its prisoners in jeopardy. When Government locks a man up it is sacredly bound to make the hygienic conditions of his incarceration as good for him as prison life allows. There can be no good excuse for doing less than this. When a prison is filthy, so crowded, so ill-fed, or so cruelly governed, as to make the breeding of disease in its cells a certainty, sin lies at the door. No Government that has a right to imprison men is so poor that it cannot provide reasonably for their health. The best conducted prison might be expected to show a higher death-rate than the average of free life. When the prison death-rate is exceptionally large it is the evidence of negligence or oppression. The Government that neglects or oppresses its prisoners is guilty of an unpardonable sin. It makes punishment persecution, and justice vengeance."

The matters of hygiene referred to in the discussions were interesting, but disclosed no advanced suggestions or principles not already known to the professional reader. Experienee has shown, what, indeed, common-sense ought to dictate, that an average supply of substantial, but not luxurious, food, a plenty of room, good ventllation, good water, and sufficient warmth to prevent suffering in the winter, are among the requisites to ordinary comfort and health, and ought not to be denied the prisoner.

The point so frequently urged, that efforts for the moral reformation of the prisoner should not be withheld, is wise and proper; and yet, with all these, it must not be forgotten that the crim'nal deserves punishment, and that he is the subject of Justice rather than of Mercy.

